# Synthesis and Characterization of Calcium Phosphate Materials Derived from Eggshells from Different Poultry with and without the Eggshell Membrane

**DOI:** 10.3390/ma15030934

**Published:** 2022-01-26

**Authors:** Marta Kalbarczyk, Aleksandra Szcześ, Izolda Kantor, Zoltan May, Dariusz Sternik

**Affiliations:** 1 Department of Interfacial Phenomena, Faculty of Chemistry, Institute of Chemical Sciences, Maria Curie-Sklodowska University in Lublin, M. Curie-Sklodowska Sq. 3, 20-031 Lublin, Poland; marta.kalbarczyk@poczta.umcs.lublin.pl; 2 Research Centre for Natural Sciences, Plasma Chemistry Research Group, Institute of Materials and Environmental Sciences, Magyar Tudósok Krt. 2, H-1117 Budapest, Hungary; kantor.izolda@ttk.hu (I.K.); may.zoltan@ttk.hu (Z.M.); 3 Research Institute of Biomolecular and Chemical Engineering, University of Pannonia, Egyetem u. 10, H-8200 Veszprém, Hungary; 4 Department of Physical Chemistry, Faculty of Chemistry, Institute of Chemical Sciences, Maria Curie-Sklodowska University in Lublin, M. Curie-Sklodowska Sq. 3, 20-031 Lublin, Poland; dariusz.sternik@mail.umcs.pl

**Keywords:** hydroxyapatite, calcium phosphate, eggshells, the eggshell membrane

## Abstract

Calcium phosphate materials such as hydroxyapatite (HA) or tricalcium phosphate (β-TCP) are highly attractive due to their multitude of applications in bone replacement as well as their environmental and ecological credentials. In this research, quail, hen, duck, and pigeon eggshells were used as a calcium source to obtain calcium phosphate materials via the environmentally friendly wet synthesis. Using the eggshells with the organic membrane, the biphasic calcium phosphate materials composed mainly of HA were obtained. The second mineral phase was β-TCP in the case of using quail, hen, and pigeon eggshells and octacalcium phosphate (OCP) in the case of duck eggshells. The HA content in the obtained materials depended on the amount of membrane in the eggshells and decreased in the order of pigeon, duck, hen, and quail eggshells. The eggshell membrane removal from the eggshells caused the reduced content of HA and the presence of the more soluble β-TCP or OCP phase in the obtained materials. The calcium ions release profile in the PBS buffer indicates the potential biomedical application of these materials.

## 1. Introduction

The biphasic calcium phosphates (BCPs) are bioceramics composed of two phases at different ratios, mostly hydroxyapatite (HA) and β-tricalcium phosphate (β-TCP) [1,2]. By changing the HA/β-TCP ratio in the mixture, the biological properties can be optimized compared to the monophasic calcium phosphates [3,4,5] due to the balance between the resorption and solubilization processes [6]. Moreover, the grain size, crystallinity, porosity, pore size, and presence of trace elements can affect biological reactivity [5,7,8]. The wide range of applications of those composites are connected with not only the bone and dental replacements but also the possibility of the BCP use as a drug carrier or a fertilizing agent [9,10,11,12].

The preparation of biphasic and multiphasic calcium phosphates is mainly proceeded by the high-temperature (higher than 750 °C) sintering of non-apatitic calcium phosphates, such as amorphous calcium phosphate (ACP) or calcium deficient hydroxyapatite phosphates (CDHA) as well as using the two-step sintering process with microwave irradiation. The validity of employing other techniques was also proved, i.e., solid state reaction [13], flame spray pyrolysis [14], liquid mix, sol–gel method, hydrothermal treatment, or high-temperature combustion synthesis [3]. All the above mentioned methods require the application of high temperature which is less favorable due to the economic and environmental considerations [3]. The BCP material can also be obtained via mechanical mixing [3]; however, it was proved that the composite was less stable during sintering. As a result, the HA/β-TCP ratio changed with the high temperature treatment [15] and a third α-TCP phase also reportedly appeared [16].

Due to economic and environmental considerations, there is still a growing interest of replacing synthetic reagents with the recycled ones, originated from wastes or residues. Using waste resources as the precursors during the chemical synthesis is thus a modern and beneficial approach. According to the recent literature, there are a few effective methods of the hydroxyapatite synthesis from wastes such as various animal bones (e.g., bovine bones [17], fish scales [18], algae [19], corals [20], shells [21], or eggshells [22,23]). The materials obtained in this way are composed mostly of HA [17,18,21,22]; however, the products containing considerable amounts of a second phase, in most cases β-TCP, were also described [10].

Eggshells were used to synthesize BCP. Ho et al. [24] obtained the product composed mainly of HA with a significant amount of β-TCP from the raw eggshells without the inner membrane in the solid state reaction with the DCPD (dicalcium phosphate dehydrate). The prepared mixture was heated for 1, 3, or 5 h in the temperature range 800–1150 °C. Extending the high temperature exposure time resulted in the decrease of the β-TCP amount as well as the disappearance of the reagents. Ganesan et al. [25] obtained the mesoporous BCP material from the calcined eggshells using the rapid thermal processing (RTP). The results showed that the pure HA material could be transformed into the BCP composed of ca. 60% HA and 40% β-TCP by heating at 1150 °C. It was also pointed out that the thermal treatment extension resulted in a larger content of β-TCP in the obtained material, whereas the surface area was larger for the samples exposed to the lower temperature. The BCP material composed of HA and β-TCP phases was synthesized by Wu et al. [26] using powdered oyster eggshells rich in DCPD (dicalcium phosphate dehydrate) and stripped of the membrane as a starting material, through ball milling and then subsequent heat treatment at 1000 °C. The ball-milling time extension caused a decrease of the β-TCP content in the tested samples. Zanelato et al. [23] obtained material composed of two phases: 85% HA and 15% DCPA during the wet precipitation synthesis procedure using calcined hen eggshells as a precursor of calcium ions and 85% phosphoric acid. Mohd Pu’ad and coworkers [27] used boiled and calcined eggshells to obtain HA by means of wet chemical precipitation methods. The material was sintered at higher temperature ranging from 300 °C to 1100 °C. The formation of the BCP composed of HA and β-TCP was evident at temperatures higher than 700 °C.

In recent years, not only eggshells, but also eggshell membranes (ESM) are gaining more interest as biowastes that can be used in material science [28]. Approximately 80–85% of ESM fibers are proteins, 10% of which are collagen types I, V, and X, and 70–75% of other glycoproteins and proteins [29]. The ESM consists of three layers: the outer ESM, the inner ESM and the limiting membrane (LM) of different thickness. While the outer eggshell membrane is firmly attached to the eggshell, the other two parts can be mechanically separated [30].

To our knowledge, there are few studies showing the effect of ESM on the crystallization of calcium phosphates. Recently [31] eggshell membranes were used as a bio-template for microwave-synthesized HA. The presence of crystallized hydroxyapatite and triclinic monetite was confirmed on the impregnated ESM surface. Moreover, the size of the resulting HA was not uniform. Spherical agglomerates with a diameter of a few microns along with bigger structures with size up to 15 µm were obtained. Zhang et al. [32] obtained the flower-like hydroxyapatite agglomerates on the upper and lower side of an eggshell membrane peeled off from the hen egg. It was found that a higher temperature, moderated holding time and a higher pH value promoted the formation of the flower-like hydroxyapatite agglomerates with high crystallinity.

As shown in the literature, BCP has been obtained with the inclusion of the sintering process at elevated temperatures. The scope of our research was to obtain the BCP material using wastes in the form of eggshells as a calcium source. In previous papers, the synthesis of biphasic and multiphasic calcium phosphate materials at different pH using hen eggshells has been described [33,34]. An element of the novelty is the reduction of energy consumption through the elimination of the sintering process during the synthesis. Moreover, eggshells of various poultry species with and without the eggshell organic membrane were used, allowing the impact of the membrane on the composition and morphology of the obtained composites to be evaluated for the first time. Furthermore, the calcium ion release profiles of all the obtained composites were determined, pointing to their potential biomedical applications.

## 2. Materials and Methods

### 2.1. Materials

All reagents, i.e., HNO_3_, Na_2_HPO_4_, NaOH, and EtOH, were pure per analysis (ppa) grade and purchased from AVANTOR S.A. (Gliwice, Poland). The standard solutions were used for the inductively coupled plasma optical emission spectroscopy (ICP-OES) and inductively coupled plasma mass spectroscopy (ICP-MS) instruments calibration: CPAChem Ltd. (Stara Zagora, Bulgaria) multi-element standard solution (33 elements, M8A96.K1.5N.L5) and Spectrascan multi-element standard solution for rare earth elements (D2-MEB359019). The Phosphate Buffer Solution (PBS) tablets were purchased from Sigma Aldrich (St. Louis, MO, USA) and dissolved in water according to the producer’s recommendations. The water used for the solutions preparation was purified with the Milli-Q Plus system (Millipore, Bedford, MA, USA) with a resistivity of 18.2 MΩ cm^−1^ and a total organic content (ToC) <4 ppm. The hen, pigeon, and duck eggshells were collected from the households near Lublin (Poland) while the quail eggs were acquired from a local organic food store. All the eggshells were divided into two categories. In the first, the eggshells were thoroughly washed with water, dried at 50 °C and crushed into fine powders, whilst for the second category, the eggshells were rinsed with water, peeled from the organic membrane, boiled in MilliQ water for 15 min to remove organic residues, rinsed again with water, dried and grated. The prepared materials were analyzed to quantify their organic residue content using the thermogravimetric techniques and the element composition using the ICP-OES (calcium and magnesium content) and ICP-MS (copper and zinc content) methods. For the elemental analysis of the eggshell samples, 100 mg of each sample was weighed with analytical balance into glass beakers and 10 mL of concentrated nitric acid (67%, AR—the standard Macron Fine Chemicals™ grade of analytical reagents) was then added to each sample and covered with watch glasses. After this, all the samples were put on a hot plate, heated and boiled between 100 and 150 °C about for 4 h in order to digest the samples completely. After cooling, the solutions were poured into 10 mL volumetric flasks and completed with ultrapure water and were measured with ICP-OES. For the ICP-MS measurements the sample stock solutions were diluted by a factor of ten.

### 2.2. Synthesis Procedure

The synthesis of biphasic materials from the eggshells was carried out according to the procedure described previously [34]. The same amounts of each eggshell sample were weighed, dissolved in 15 mL of 1 M nitric acid (the amounts of eggshells were calculated from the DSC results) and stirred for one hour to complete the dissolution of the shells. The appropriate stoichiometric volumes (the molar ratio of calcium to phosphorus was 1.67) of 0.3 M Na_2_HPO_4_ were added dropwise using a peristaltic pump with a constant flow rate of 20 mL min^−1^. The pH of each mixture was adjusted to 11.4 using the 1 M NaOH solution. After 4 h stirring, the suspensions were kept in the furnace at 50 °C for 24 h, then filtered with the Buchner funnel, washed with ultrapure water and EtOH, and dried in the furnace at 50 °C.

### 2.3. Characterization of the Obtained Materials

To evaluate the elemental composition of the prepared eggshell samples, the inductively coupled plasma optical emission spectroscopy (ICP-OES) and inductively coupled plasma mass spectroscopy (ICP-MS) techniques were applied. These stock solutions were measured directly with a simultaneous ICP-OES spectrometer with axial plasma viewing (Spectro Genesis, Kleve, Germany) and quadrupole iCAP Q ICP-Mass Spectrometer (Thermo Scientific, Waltham, MA, USA) with the KED mode (Kinetic Energy Discrimination) using ultrapure helium gas.

The mineral phase composition was determined using an X-ray PANalytical Empyrean diffractometer (Malvern PANalytical, Malvern, UK) with the PIXcel-3D detector using monochromated Cu–Kα radiation (λ = 1.54184 Å), 40 kV and 35 mA, in the 2θ range of 4.1−60°, with an acquisition step of 0.02°, and an acquisition time of 100 s. To identify crystalline phases, the HighScore Plus 3.0e (3.0.5) software supplied by PANalytical (Almelo, The Netherlands) basing on the ICDD PDF4 + 2019 diffraction database was used. The composition of the products was determined by the Rietveld method [35,36] through fitting the obtained patterns to the diffractograms from the database using producer’s software.

The morphology and size of the crystalline products were determined using the scanning electron microscopy (DualBeam Quanta 3D FEG, FEI, Hillsboro, OR, USA) with Everhart-Thornley detector (ETD) at the accelerating voltage equal to 30 kV under the high vacuum condition. Small amounts of the powders fixed on the stubs with carbon tapes and coated with gold layer by vacuum sputtering were measured.

The thermal analysis was made using a STA 449 Jupiter F1 apparatus (Netzsch, Selb, Germany) coupled with an IR spectrometer in the synthetic air atmosphere at the heating rate of 10 °C min^−1^ in the temperature range from the room temperature to 1100 °C.

### 2.4. Calcium Ions Release Profiles

To determine the calcium ion release at 24 °C ± 1, the samples of each composite were soaked in the PBS buffer (pH = 7.4) at the concentration of 1 mg mL^−1^. After the chosen time periods, the supernatant samples were collected with syringes, filtered with 0.22 µm PTFE hydrophilic syringe filters (AlfaChem, Poznań, Poland) and diluted at the ratio 1:4 with ultrapure water. The prepared samples were analysed using the ICP-OES technique due to the calcium content in the supernatant.

## 3. Results and Discussion

### Eggshells Samples Characterization

**Figure 1:**
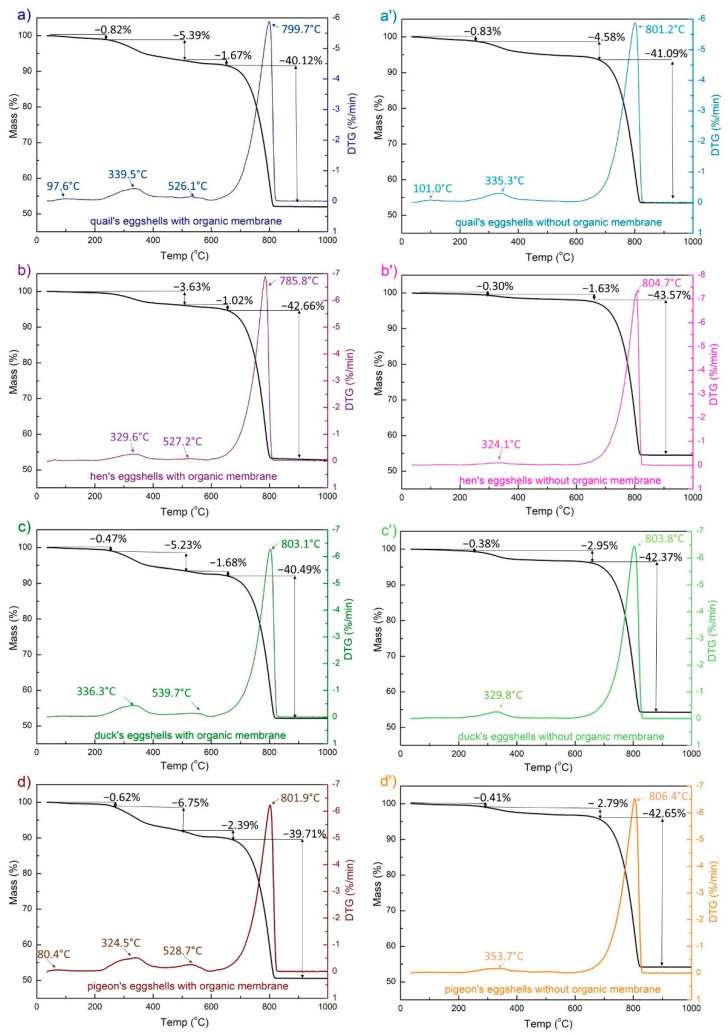
Results from the thermal analysis: TG (black) and DTG (colored) curves of different poultry species eggshell materials with (**a**–**d**) and without (**a’**–**d’**) the eggshell membrane (EMS).

The eggshells used for the synthesis as calcium sources were studied by means of the thermogravimetric method (Figure 1). An endothermic peak of about 800 °C, characteristic of the calcium carbonate decomposition, was present in all the samples. Compared to the eggshells containing the membrane (Figure 1a–d), for all the samples without an eggshell membrane (Figure 1a’–d’), the peak is shifted a few degrees towards the higher values. The weight loss observed in the two temperature ranges, i.e., 200–450 °C and 450–600 °C can be attributed to the lattice water and the organic membranes degradation. For all the eggshells containing ESM, the two peaks appear on the DSC and DTG curves at ~340 °C and 540 °C. The degradation peak at the higher temperature may be due to the strong interactions of the organic component and CaCO_3_ [37]. When the membranes are removed from the eggshell, the latter disappears (Figure 1a’–d’). The small endothermic peaks around 100 °C for the quail eggshells with and without an ESM (Figure 1a,a’) and the pigeon eggshells with EMS (Figure 1d) are associated with the water loss. The eggshells containing the eggshell membrane decomposed in the temperature range from 200 to 600 °C with 4.65%, 7.38%, 7.88%, and 9.76% mass loss, for hen, duck, quail, and pigeon eggshells, respectively.

**Figure 2:**
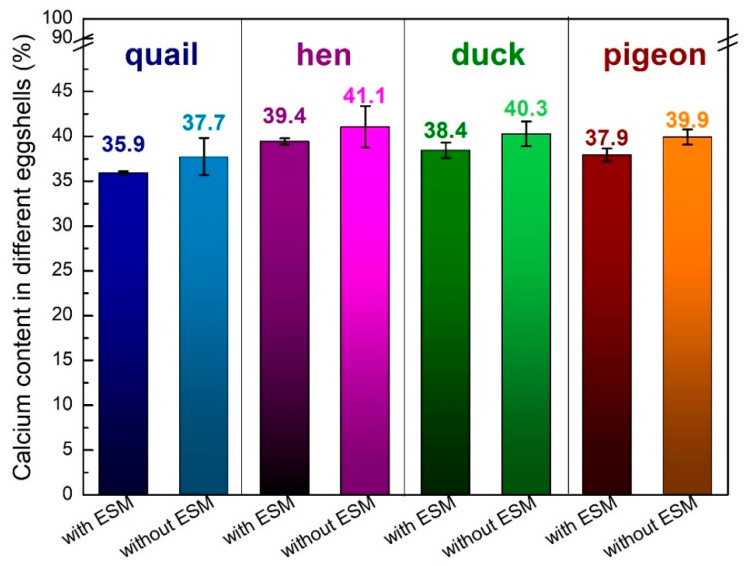
Calcium content (weight %) in the various poultry species eggshells with and without the eggshell membrane determined using the ICP-OES techniques.

**Figure 3:**
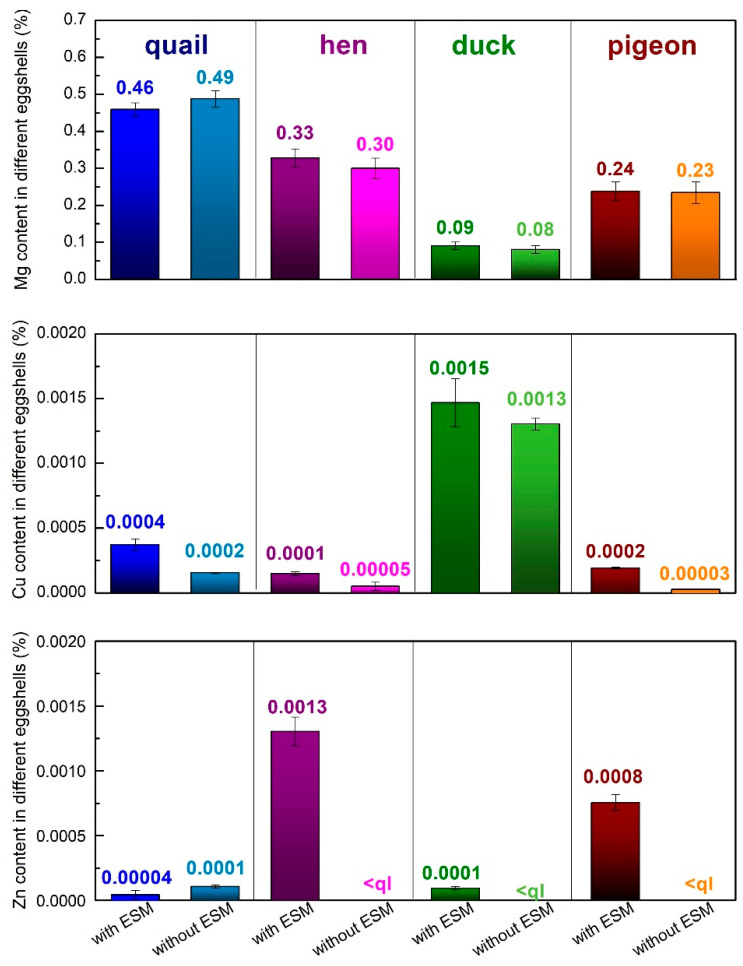
Magnesium, copper, and zinc contents (*w*/*w*%) in various poultry species eggshells determined with the ICP-OES (magnesium) and ICP-MS (Copper and zinc measurements (<ql indicates below the quantification limit; for Zn, ql = 4.94·10^−6^%).

The elemental composition of each eggshell was determined using the ICP-OES techniques (Figure 2 and Figure 3). Each shell was composed mostly of calcium (around 40% *w*/*w*), magnesium (less than 0.5% *w*/*w*) and tiny amounts of copper and zinc. After the organic membrane removal, the calcium content increased by about 2%, which is in line with the results of the thermal analysis. The smallest amount of this element was detected for quail eggshells, whereas the largest one for the hen eggshell samples.

Significant changes in the composition were observed during other metals ion content analyses (Figure 3). The largest magnesium content was found in the eggshells of quail (about 0.5%) and only a tiny amount, less than 0.1%, of this element was detected in the duck eggshells. It is also worth mentioning that only in the quail eggshell the magnesium amount increased after the eggshell membrane removal. In other eggshells, a small decrease of magnesium content was observed after peeling off the ESM. Trace amounts of copper and zinc were also detected. The duck eggshells contained the highest amount of copper while the hen eggshells contained a larger amount of zinc in comparison to the other samples. All the analyzed samples showed the tendency toward a decrease in both metal ions after the EMS removal which indicates that the presence of those metals is strongly related to the organic part of the eggshell.

**Figure 4:**
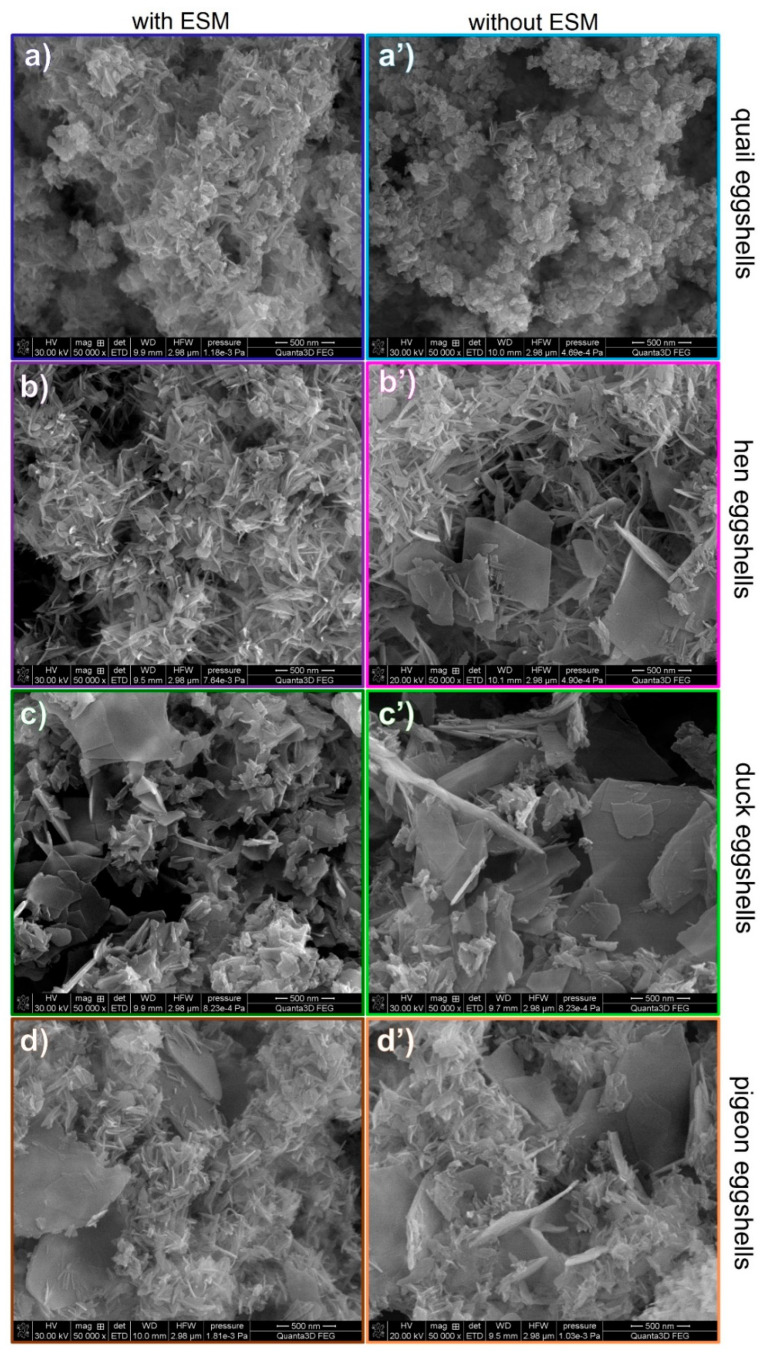
SEM images of the materials derived from different poultry eggshells with (**a**–**d**) and without (**a’**–**d’**) an EMS.

The SEM images in Figure 4 show that the crystal morphology and size the obtained calcium phosphate materials depend on the eggshells type. The material obtained from the quail eggshells was composed of thin and short rod-shaped crystals as well as small hexagonal flakes of a few nanometers in size (Figure 4a). In the case of the products derived from the hen eggshells, needle-like aggregates were observed (Figure 4b). Moreover, the small hexagonal flakes emerged. The use of duck and pigeon eggshells as a calcium biosource resulted in more diverse structures (Figure 4c,d). All these samples were composed of short nanorods as well as sheet-like forms, sparsely interspersed by hexagonal flakes. When the pigeon eggshells were used for the synthesis, the nanorod shaped crystals were mostly observed and they were of smaller size (Figure 4d) compared to the products derived from the hen eggshells, while in the product obtained from the duck eggshells there was no prevailing structure (Figure 4c).

Removing the EMS affected the shape and size of the obtained crystals irrespective of the type of the eggshells used. The products from the quail eggshells exhibited the cauliflower-like aggregates structure but composed of small hexagonal nanoflakes (Figure 4b’). In the case of the products derived from the hen eggshells, significant changes of the crystals shape were observed (Figure 4b’). When the eggshells were purified from the organic membrane, two types of shapes were observed: sheets with the width and length about 500 nm and much smaller nanorods. When the pigeon and duck eggshells were used for the synthesis after the ESM removal, the sheet-shaped structures became more widespread (Figure 4c’,d’) compared to the products when the eggshells with the organic residues were processed. It can also be seen that the sheets were larger when the eggshells without organic residues were used.

Spherical “flowers” agglomerates were obtained at pH = 11.0 on the upper and lower side of the hen eggshell membrane by Zhang et al. [32]. A mixture of agglomerates with the spherical morphology of a diameter a few microns and bigger structures with dimensions up to 15 µm were obtained on the EMS template [31]. It seems that the organic material would enhance the nucleation rate and crystal growth in a certain direction. Therefore, smaller crystals with elongated shapes tend to form in the presence of ESM.

**Figure 5:**
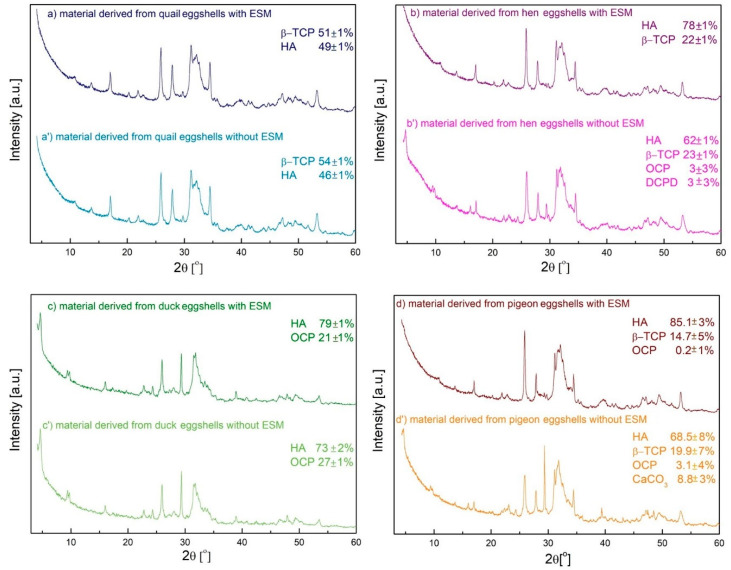
XRD results of the materials derived from different poultry species eggshells with (**a**–**d**) and without (**a’**–**d’**) EMS; (HA: (ICDD) 04-010-6312, β-TCP: (ICDD) 04-001-7220, OCP: (ICDD) 00-044-0778, DCPD: (ICDD) 01-072-1240, CaCO_3_: (ICDD) 04-012-8072).

The composition of the obtained materials was investigated with the XRD technique and analyzed by the Rietveld method (Figure 5). This method is based on the best fit of the experimental diffractogram to the theoretical one, using the least square method. Instead of a few peaks of the highest intensities, a full diffraction pattern is used for calculation [35,36].

All the samples obtained from the eggshells containing the organic membrane were composed of two forms: HA and a second one depending on the egg types. The HA was the main component and its percentage content decreased from 85.1% to 49% in the order of pigeon, duck, hen, and quail eggshells. It is worth noticing that, in the case of duck and hen eggshells with the membrane, the HA content was almost the same. These trends correspond with those in the content of organic substances determined using the thermogravimetric analysis (cf. Figure 1). The samples derived from various poultry species with the EMS were diverse in terms of the second phase. With the exception of the material obtained from the duck eggshell which contained OCP, the second phase was β-TCP for all the other materials. This may be due to the lowest magnesium content in the duck eggshells. In the case of pigeon eggshells a small amount of OCP appeared, however, within the calculation error (the characteristic peak at 4° was not visible).

Only a slight impact of the organic matter was observed for the biphasic materials obtained from the quail and duck eggshells. In comparison with the materials obtained from the shells with EMS, the HA content decreased by 3% in the former and by 6% in the latter. The materials derived from the hen eggshells with an organic membrane were composed of 78% of HA and 22% of β-TCP whereas the use of the eggshells without the EMS caused the appearance of two less stable forms: OCP and DCPD with the significantly reduced HA content and the same β-TCP content. In the case of the material derived from the pigeon eggshells with the organic residues, the largest content of HA was observed (Figure 5d) and a secondary phase was β-TCP (14.7%), with tiny amounts of OCP also detected (0.2%). When the peeled pigeon eggshells were applied as a calcium source, the HA and β-TCP content decreased whereas the OCP content increased from 0.2% to 3.1%. Moreover, the presence of calcium carbonate in the analyzed sample indicates that the eggshells were not completely dissolved in the acid during the synthesis.

It can be seen from the presented results that, using different type of eggshell with EMS, it is possible to obtain biphasic calcium phosphate materials of different compositions. The composition clearly depends on the type of eggs used. Quail, hen, and pigeon eggshells yielded material consisting of β-TCP and HA in an approximate ratio of 1:1; 1:3.5, and 1:6, respectively. In the case of duck eggshells, the material obtained consisted of OCP and HA in ~1:4 ratio. Biphasic calcium phosphate mixtures containing HA and β-TCP or DCPD were obtained previously using eggshells without EMS or calcined ones [25,26,27] and sintered at the high temperature. In contrast, the energy consumption has been greatly reduced in our research. The mechanical removal of EMS from the eggshells reduced the content of the most stable phase (HA) of calcium phosphate at pH = 11.4 and the content of more soluble forms (β-TCP, OCP, and DCPD) increased. It is conceivable that the organic EMS containing functional groups derived from amino acids could increase the local concentration of ions and accelerate the transformation of less stable calcium phosphate forms into more stable HA. This is consistent with the observation that increasing the nucleation rates led to the formation of a larger number of smaller crystals.

**Figure 6:**
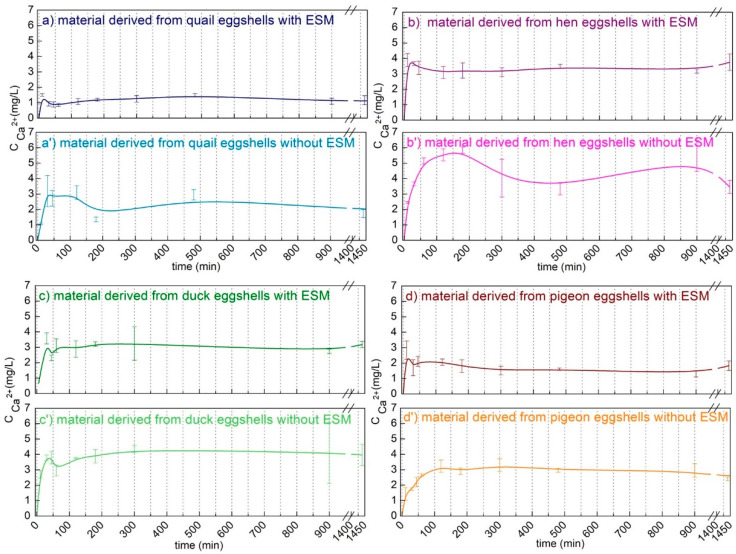
Calcium ions release profiles in the PBS buffer of the materials derived from different poultry species eggshells with (**a**–**d**) and without (**a’**–**d’**) an eggshell membrane (EMS).

To demonstrate the potential biomedical use of the obtained calcium phosphate materials, the release of calcium ions were determined in the PBS buffer at pH 7.4 at room temperature. As shown in Figure 6, most calcium was released in the first hour. In the case of the material obtained from the eggshells containing a membrane, the concentration of the released calcium ions in the solution decreased in the order hen > duck > pigeon > quail. However, the ion concentrations released from the materials obtained from hen and duck eggshells were only slightly different. In the case of calcium phosphate obtained from the eggshells without the organic membrane, the concentration of the released calcium ions increased. Moreover, the profile of the concentration changed over time indicating that the release process of these ions was slower. This was the least noticeable in the case of duck eggshells. The higher the content of the least soluble form, i.e., HA, the lower the release of calcium ions. The results are consistent with the XRD data (Figure 5), showing a smaller content of the most stable HA in the materials obtained from the eggshells without the organic membrane. The presence of the second, more soluble phase in the product can provide the crucial reagents for the biomineralization process (that is, calcium and phosphate ions), while stable HA can act as a scaffold of the hard tissue replacement material.

## 4. Conclusions

In this work, a simple and environmentally friendly method has been applied to convert the agricultural wastes (eggshells) into the mainly biphasic calcium phosphate material mixture composed of HA and β-TCP or OCP. The materials obtained from quail, hen, and pigeon eggshells with EMS consisted of β-TCP and HA in an approximate ratio of 1:1, 1:3.5, and 1:6, respectively. In the case of duck eggshells, the material obtained consisted of OCP and HA in approximately 1:4 ratio. The presence of OCP as the second major component in the material obtained from the duck eggshell may be due to the low magnesium content in the eggshells. The organic membrane removal from the eggshells resulted in a larger content of the more soluble β-TCP or OCP phases which was also reflected in the higher concentration of the released calcium ions. The calcium ions release profile demonstrates the potential biomedical application of the obtained materials which will motivate further research for their specific usage.

## Data Availability

Not applicable.
